# Efficacy and safety of pyrotinib combined with albumin‐bound paclitaxel as first‐line treatment for HER2‐positive metastatic breast cancer in patients previously treated with adjuvant and/or neoadjuvant trastuzumab therapy: The stage 1 results of a single‐arm, phase 2 prospective clinical trial

**DOI:** 10.1002/ctm2.1687

**Published:** 2024-05-13

**Authors:** Xiaochu Man, Jie Huang, Shujuan Sun, Dongdong Zhou, Baoxuan Zhang, Shu Fang, Fangchao Zheng, Chao Li, Xinzhao Wang, Wei Huang, Linlin Wang, Qingqing He, Hui Fu, Yan Zhang, Changrui Liu, Lin Dong, Xianguang Zhao, Liang Xu, Xiao Sun, Bingjie Fan, Lihua Song, Zhengbo Zhou, Jinming Yu, Huihui Li

**Affiliations:** ^1^ Department of Breast Medical Oncology, Shandong Cancer Hospital and Institute Shandong First Medical University and Shandong Academy of Medical Sciences Jinan China; ^2^ Department of Breast Surgery, Shandong Cancer Hospital and Institute Shandong First Medical University and Shandong Academy of Medical Sciences Jinan China; ^3^ Department of Radiation Oncology, Shandong Cancer Hospital and Institute Shandong First Medical University and Shandong Academy of Medical Sciences Jinan China; ^4^ Department of Surgery The 960th Hospital of the PLA Joint Logistics Support Force Jinan China; ^5^ Department of Medical Oncology Qingdao Municipal Hospital (Group) Jinan China; ^6^ Department of Surgery Liaocheng Tumor Hospital Liaocheng China; ^7^ Department of Radiology, Shandong Cancer Hospital and Institute Shandong First Medical University and Shandong Academy of Medical Sciences Jinan China

**Keywords:** albumin‐bound paclitaxel, HER2‐positive metastatic breast cancer, Olink, Pyrotinib

## Abstract

**Objective:**

It has been observed that the prognosis of patients with HER2‐positive metastatic breast cancer has improved significantly with HER2‐targeted agents. However, there is still a lack of evidence regarding first‐line anti‐HER2 treatment options for patients who have received adjuvant and/or neoadjuvant trastuzumab for HER2‐positive metastatic breast cancer. Besides, there are no reliable markers that can predict the efficacy of anti‐HER2 treatment in these patients.

**Methods:**

Patients who have received adjuvant and/or neoadjuvant trastuzumab for HER2‐positive metastatic breast cancer were enrolled. Pyrotinib plus albumin‐bound paclitaxel were used as first‐line treatment. The primary endpoint was the objective response rate (ORR). The safety profile was also assessed. In order to explore predictive biomarkers using Olink technology, blood samples were collected dynamically.

**Results:**

From December 2019 to August 2023, the first stage of the study involved 27 eligible patients. It has not yet reached the median PFS despite the median follow‐up being 17.8 months. Efficacy evaluation showed that the ORR was 92.6%, and the DCR was 100%. Adverse events of grade 3 or higher included diarrhoea (29.6%), leukopenia (11.1%), neutropenia (25.9%), oral mucositis (3.7%), and hand‐foot syndrome (3.7%). Toll‐like receptor 3 (TLR3) and Proto‐oncogene tyrosine‐protein kinase receptor (RET) were proteins with significant relevance to PFS in these patients.

**Conclusions:**

This study demonstrates that pyrotinib plus albumin‐bound paclitaxel as a first‐line treatment regimen shows good efficacy and manageable safety for patients who have received adjuvant and/or neoadjuvant trastuzumab for HER2‐positive metastatic breast cancer. Besides, a significant association was identified between the expression levels of TLR3 and RET and the PFS in patients.

## Graphical abstract

The combination of pyrotinib and albumin‐bound paclitaxel as a first‐line treatment regimen delivers favorable efficacy with manageable safety in patients with HER2‐positive metastatic breast cancer who have previously received adjuvant and/or neoadjuvant trastuzumab therapy. And TLR3 and RET might serve as potential predictive proteins for progression‐free survival in these patients.

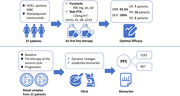

## 1 BACKGROUND

Overexpression of human epidermal growth factor receptor 2 (HER2) is observed in 25−30% of patients with breast cancer and has been associated with a poorer prognosis.^1^, ^2^ In patients with breast cancer that is HER2‐positive, targeted therapies have significantly improved the prognosis.^3^ Trastuzumab combined with chemotherapy remains the primary choice for HER2‐positive breast cancer. However, the development of trastuzumab resistance due to alterations in active target receptors or downstream components of the PI3K/Akt/mTOR signalling pathway is inevitable. Thus, novel anti‐HER2 strategies must be developed to provide alternative treatments for patients who become resistant to standard therapies. Although trastuzumab, pertuzumab, plus docetaxel were found to extend overall survival (OS) among patients with HER2‐positive breast cancer as a first‐line therapy was observed in the CLEOPATRA study, only 11% of the study population had received trastuzumab.^4^ The Nefertiti trial, which investigated the efficacy of neratinib, a small molecule tyrosine kinase inhibitor (TKI), plus paclitaxel compared to trastuzumab plus paclitaxel as a first‐line treatment regimen in patients with HER2‐positive metastatic breast cancer, yielded an unfavorable outcome, as evidenced by an objective response rate (ORR) of 74.8% in the neratinib cohort, falling short of the 77.6% observed in the trastuzumab cohort, and only 11.6% of patients had received prior trastuzumab treatment in the neratinib cohort.^5^ The PHILA study showed that the addition of pyrotinib to trastuzumab and docetaxel resulted in a significant improvement in progression‐free survival, however only 14−15% of patients had previously received trastuzumab in the neoadjuvant or adjuvant setting.

Pyrotinib is a second‐generation, irreversible pan‐ErbB receptor targeting EGFR, HER2, and HER4.^6^ Several studies have shown favourable efficacy of pyrotinib in the treatment of advanced breast cancer that is HER2‐positive.^7^, ^8^, ^9^ Additionally, albumin‐bound paclitaxel (nab‐PTX), a commonly used chemotherapeutic, has also been demonstrated favourable efficacy in combination with carboplatin and trastuzumab in the treatment of advanced breast cancer that is HER2‐positive.^10^ However, conclusive studies on nab‐PTX plus pyrotinib as first‐line treatment for patients with HER2‐positive metastatic breast cancer who have received adjuvant and/or neoadjuvant trastuzumab therapy are lacking.

Several studies have shown that the dynamic changes in protein markers are associated with the treatment efficacy or patient prognosis.^11^, ^12^ Currently, proteomics detection relies heavily on mass spectrometry, but low levels of target molecules may be difficult to detect in complex environments. Olink technology has become increasingly popular in recent years for proteomic analyses. Several studies have utilized the Olink technology for prognostic‐related analyses and have achieved outstanding outcomes.^13^, ^14^ In contrast to conventional proteomics methods, Olink technology has demonstrated improved specificity and sensitivity in the analysis of biological samples.^15^ The dynamic detection of plasma proteins during treatment enables the visual monitoring of treatment response and prediction of prognosis, facilitating the identification of the beneficiary population. Yet, there are no studies exploring protein markers to predict the efficacy of pyrotinib combination regimens and PFS of patients. Therefore, there is an urgent need to explore the predictive markers in order to aid in identifying the beneficiary population.

In this study, pyrotinib plus nab‐PTX as the first‐line treatment was evaluated for efficacy and safety for HER2‐positive metastatic breast cancer that has progressed after adjuvant and/or neoadjuvant trastuzumab therapy. Proteomics was also used to explore markers associated with PFS and adverse events, providing a foundation for further investigation into potential mechanisms and therapeutic targets.

## 2 METHODS

### 2.1 Study design

This was a single‐arm, phase 2 prospective clinical trial conducted in China. The protocol was approved by the ethics committee of Shandong Cancer Hospital. Our study adhered to the Good Clinical Practice guidelines and the Declaration of Helsinki. Written informed consent was obtained from all patients. This study was registered at ChiCTR.org.cn (ChiCTR1900027932).

### 2.2 Patients

Eligible participants ranged in age from 18 to 70 years, with a minimum 12‐week life expectancy, histologically confirmed HER2‐positive advanced breast cancer (immunohistochemistry score of 3+ or 2+ confirmed by fluorescent in situ hybridization), and not yet receiving first‐line treatment. Additionally, they had received adjuvant and/or neoadjuvant trastuzumab therapy previously, had a performance status on the Eastern Cooperative Oncology Group (ECOG) scale ranging from 0 to 1, and had sufficient organ function and bone marrow. Furthermore, according to the Response Evaluation Criteria in Solid Tumors version 1.1 (RECIST v1.1), each participant was required to have at least one measurable lesion. Patients who received anti‐cancer treatment within four weeks before enrollment, had previously received small molecular anti‐HER2 TKIs, had bone or skin metastases as the only target lesions, or had symptomatic brain metastases requiring therapy were excluded.

### 2.3 Treatment and procedures

Oral pyrotinib (400 mg once daily) plus intravenous nab‐PTX (125 mg/m^2^, on days 1 and 8 of a 21‐day cycle) was administered to patients in 21‐day cycles. It is recommended that patients receive nab‐PTX for at least six cycles. After six cycles, the decision to stop the nab‐PTX was made carefully by the physician and the patient. Pyrotinib was administered until disease progression, unacceptable toxicity, withdrawal of consent, or withdrawal by the investigator. Treatment delays or dose reductions were allowed to manage adverse events. Loperamide and montmorillonite powder were used to treat and prevent diarrhoea. A stepwise reduction in the dose of pyrotinib was allowed from 400 to 320 to 240 mg. Imaging evaluations (e.g., CT chest, CT abdomen/pelvis, brain MRI, etc.) were performed every 2 cycles based on the RECIST 1.1 criteria (central nervous system disease was also included).

### 2.4 Endpoints

The primary endpoint was ORR, with secondary endpoints including progression‐free survival (PFS), overall survival, and disease control rate (DCR). The safety profile was also assessed.

### 2.5 Sample collection and assay

Streck tubes (Streck) were used to collect blood samples at baseline, pre‐therapy of the second cycle, and disease progression. The blood samples were centrifuged and the supernatants were stored at −80°C until assayed. Plasma proteomics was assayed using proximity extension assay technology (Olink Bioscience AB). We selected the Olink Target 96 panel (Oncology‐II, Olink Bioscience AB), including 92 protein biomarkers, with results presented in Normalized Protein eXpression (NPX) values. Validation data for all assays are available on the manufacturer's website (www.olink.com).

### 2.6 Statistical analyses

Simon's two‐stage phase II optimal design was used for the statistical design. If more than 17 patients within the first 27 patients achieved complete response (CR) or partial response (PR) in the efficacy evaluation (ORR ≥ 63%) and demonstrated good safety, 67 patients would be added to the cohort. Seventy‐nine patients would be required considering a 15% dropout rate. The data were analyzed using SPSS 26 and R studio (version 4.2.1), with a significance level set at *p* < 0.05. The PFS was analyzed with the Kaplan‐Meier method. Correlation analysis was performed using Spearman and Pearson methods. Logistic regression and Cox proportional hazards models were utilized for univariate and multivariate regressions. Wilcox test was used to make the volcano plots. The unit of Olink is expressed in NPX, the mean difference is equivalent to log2 fold change in Olink. Plots were generated by using GraphPad Prism 9 and R studio (version 4.2.1).

## 3 RESULTS

### 3.1 Patients’ characteristics

**FIGURE 1:**
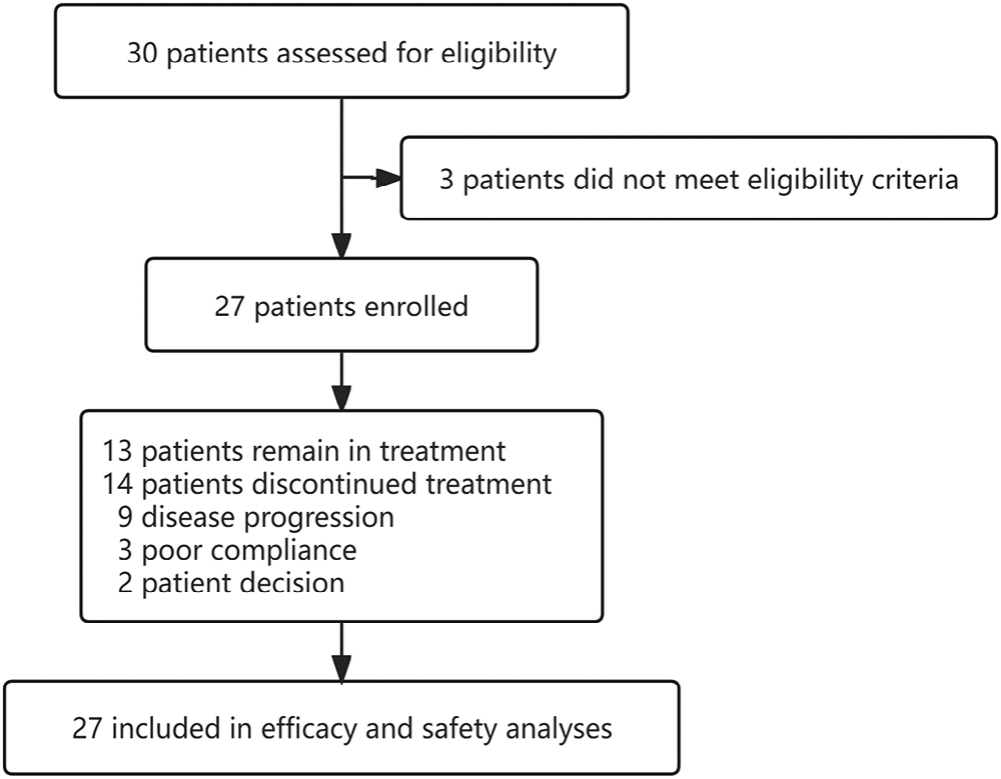
Study flowchart.

The first stage of group enrollment was completed. Thirty patients were screened for eligibility, and 27 eligible patients (median age, 53 years; range, 35–72 years) were enrolled in the efficacy and safety analyses from December 2019 to August 2023 (Figure 1). The patients' characteristics are summarized in Table 1, with 48.1% (*n* = 13) having visceral metastases; 44.4% (*n* = 12) having nonvisceral metastases; and 7.4% (*n* = 2) patients having asymptomatic brain metastases. Additionally, 40.7% (*n* = 11) were hormone receptor‐positive, with 10 of them having received adjuvant endocrine therapy. All patients have completed postoperative adjuvant chemotherapy and are scheduled to continue treatment with trastuzumab for 1 year. During the course of trastuzumab treatment, 29.6% (*n* = 8) patients failed to complete the 1‐year trastuzumab treatment, with four patients experienced disease progression after at least six months of trastuzumab treatment, and four patients discontinued treatment due to poor compliance. Twenty‐three patients received single‐target therapy (trastuzumab), while only four patients received dual‐target (pertuzumab plus trastuzumab) therapy. Based on whether more than 12 months had elapsed between the latest date of trastuzumab administration and the date of disease progression,^16^ fifteen patients were categorized into the trastuzumab primary resistance group, and 12 patients had secondary resistance.

**TABLE 1:** Baseline characteristics of patients.

Characteristics	Pyrotinib plus nab‐PTX (*n* = 27)
Age	
Median (range)	53 (35–72)
Hormone receptor status	
ER and/or PgR positive	11 (40.7%)
ER and PgR negative	16 (59.3%)
HER2 receptor status by immunohistochemistry	
2+	5 (18.5%)
3+	22 (81.5%)
Metastatic sites	
Visceral	15 (55.6%)
Brain	2 (7.4%)
Liver	3 (11.1%)
Lung	6 (14.8%)
Multi‐organ metastases	4 (55.6%)
Non‐visceral	12 (44.4%)
Previous trastuzumab therapy	
1 year	19 (70.4%)
Less than 1 year	8 (29.6%)
Single‐target or dual‐target	
Single‐target	23 (85.2%)
Dual‐target	4 (14.8%)
Resistance to previous trastuzumab	
Primary resistance	15 (55.6%)
Secondary resistance	12 (44.4%)

Abbreviations: ER, estrogen receptor; PgR, progesterone receptor.

All patients in the study were administered a minimum of six cycles of nab‐PTX therapy (median: eight cycles; range: 2–10 cycles), with the exception of one patient who withdrew consent after the second cycle. Nab‐PTX was administered to 11 patients for six cycles, nine patients for eight cycles, and five patients for ten cycles. Additionally, one patient ceased nab‐PTX therapy after nine cycles due to hand–foot syndrome.

### 3.2 Efficacy

**FIGURE 2:**
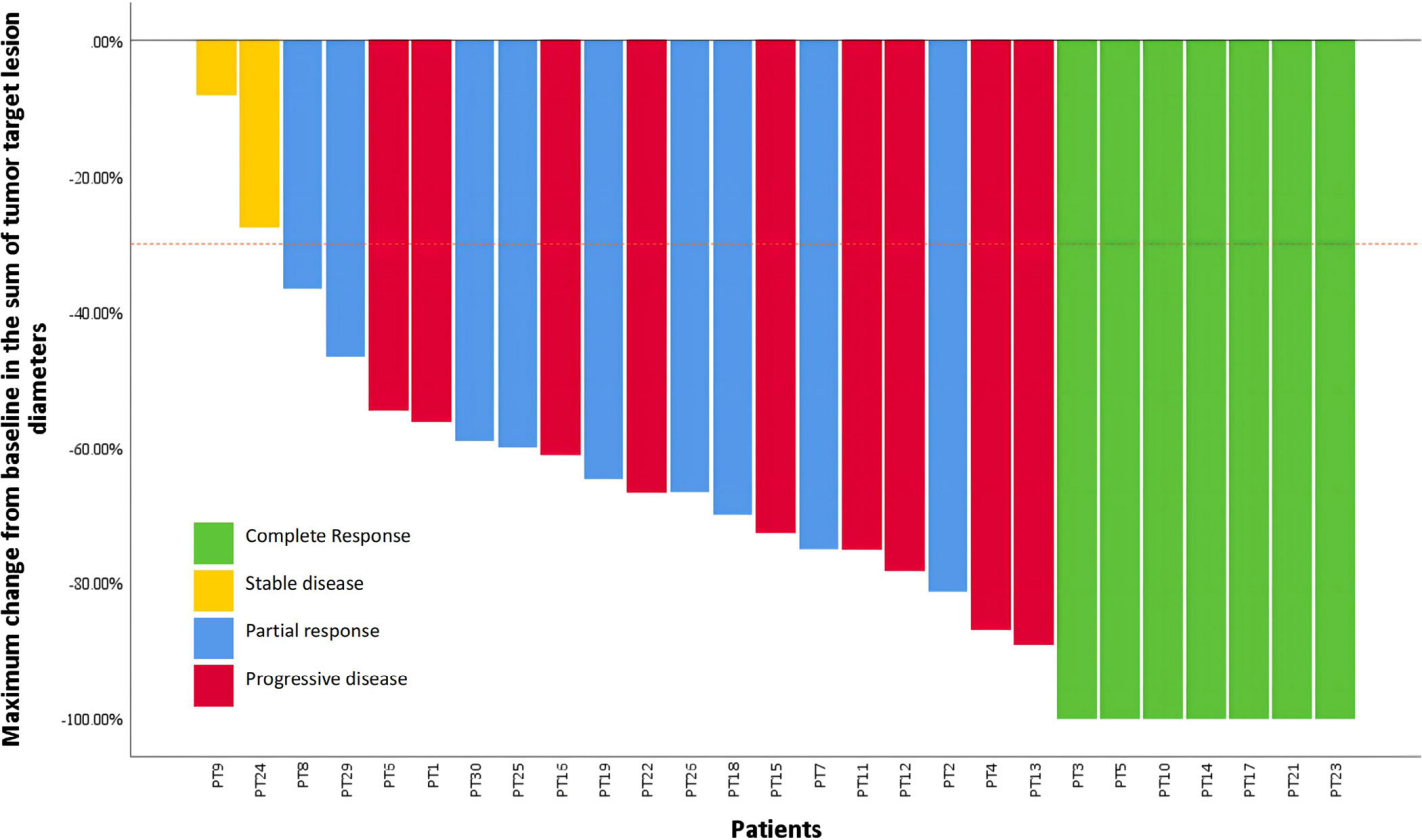
Waterfall plot for best percent change in target lesions from baseline among individual patients (*n* = 27).

As of August 2023, 27 patients were evaluated for efficacy, with seven patients achieving CR, 18 patients achieving PR, and two patients exhibiting stable disease (SD). The ORR was 92.6%, and the DCR was 100% (Figure 2). It has not yet reached the median PFS (mPFS) despite a median follow‐up of 17.8 months. As of the final follow‐up date, two patients succumbed to breast cancer. One‐year OS was 94.4%.

**FIGURE 3:**
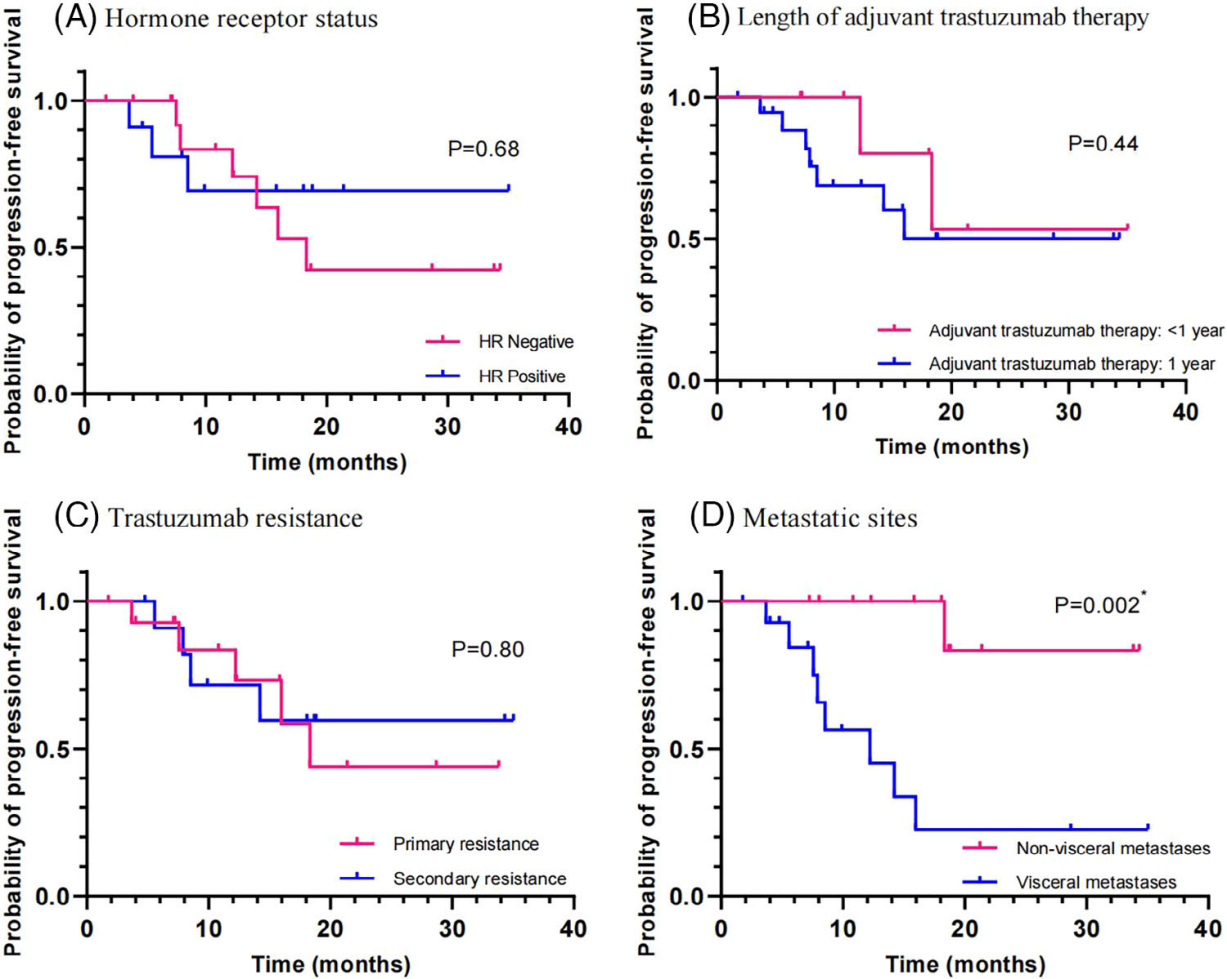
Survival analysis. (A) hormone receptor status; (B) length of adjuvant trastuzumab therapy; (C) trastuzumab resistance; (D) metastatic sites.

Subgroup analyses showed that there was no statistically significant difference between patients with hormone receptor‐positive and negative status in PFS (*p* = 0.68; Figure 3A), completion and non‐completion of 1‐year adjuvant trastuzumab therapy (*p* = 0.44; Figure 3B), primary and secondary resistance to trastuzumab (*p* = 0.80; Figure 3C). However, patients with visceral metastases had a shorter PFS than those with non‐visceral metastases (*p* = 0.002; Figure 3D).

### 3.3 Safety

Thirteen patients (48.1%) experienced treatment‐emergent adverse events (AEs) of grade 3 or higher severity, as shown in Table 2, with diarrhoea being the most frequent grade 3 AE (29.6%), followed by neutropenia (25.9%), leukopenia (11.1%), oral mucositis (3.7%), and hand‐foot syndrome (3.7%). The dose of pyrotinib was reduced in nine patients due to diarrhea, including five patients who resumed the prescribed dose of pyrotinib (400 mg once daily) after diarrhoea relief.

**TABLE 2:** Treatment‐emergent adverse events.

Adverse events	All grades	Grade 3 or higher
Any adverse events	27 (100%)	13 (48.1%)
Diarrhea	27 (100%)	8 (29.6%)
Leukopenia	25 (92.6%)	3 (11.1%)
Neutropenia	21 (77.8%)	7 (25.9%)
Anaemia	18 (66.7%)	0
Alopecia	17 (63.0%)	0
Asthenia	16 (59.3%)	0
Hand‐foot syndrome	12 (44.4%)	1 (3.7%)
Aspartate aminotransferase increased	12 (44.4%)	0
Vomiting	11 (40.7%)	0
Alanine aminotransferase increased	11 (40.7%)	0
Paresthesia	10 (37.0%)	0
Appetite loss	9 (33.3%)	0
Oral mucositis	8 (29.6%)	1 (3.7%)
Nausea	7 (25.9%)	0
Abdominal pain	6 (22.2%)	0
Weight loss	6 (22.2%)	0

The ORR of the first stage in the study met expectations (ORR = 92.6%) and adverse events were manageable. Therefore, we are going to continue enrollment in stage 2.

### 3.4 Biomarker analyses

Blood samples at baseline and pretherapy of the second cycle from 21 patients were selected for proteomic analyses. Seven of these patients also had a blood sample collected at disease progression. NPX values (Figure S1) and interquartile range (IQR) (Figure S2) were detected for analyses. One of the samples exhibited notable outliers in its distribution, leading to its exclusion from the analyses. The overall protein expression heat map is shown in Figure S3.

Dynamic changes in protein expression showed that at the pretherapy of the second cycle, 23 proteins were downregulated compared with baseline. Among them, carcinoembryonic antigen‐related cell adhesion molecule 5 (CEACAM5) showed the most significant downregulation, followed by PVRL4, ERBB2, ITGAV, and ITGB5 (Figure S4A). At the progression stage, 22 proteins were downregulated, with CEACAM5 showing the most notable downregulation, followed by PVRL4, TXLNA, and ERBB2 (Figure S4B). Throughout the entire treatment course, we observed significant downregulation of CEACAM5 and TXLNA, while PVRL4 and ERBB2 maintained significant downregulation after treatment initiation. Survival‐associated proteomic analyses revealed that Proto‐oncogene tyrosine‐protein kinase receptor (RET) and Toll‐like receptor 3 (TLR3) proteins at baseline were significantly associated with PFS (Figure S5). Further survival analyses showed that patients with high expression of TLR3 (Figure S6A) and low expression of RET (Figure S6B) at baseline had longer PFS. Compared with patients with diarrhoea grade I and II, patients with grade III diarrhoea had six proteins upregulated at baseline, with Cornulin (CRNN) being the most significant protein (Figure S7).

## 4 DISCUSSION

Significant advancements have been made in anti‐HER2 therapy since the development of trastuzumab.^17^ With more anti‐HER2 drugs becoming available, there is a need to explore combination treatment strategies and identify treatment‐related biomarkers. In this phase II study, pyrotinib plus nab‐PTX as a first‐line treatment regimen achieved an ORR of 92.6%. Despite the smaller number of patients enrolled, these results indicate that this treatment option shows promise.

Multiple studies have investigated treatment strategies following trastuzumab. Both Phase I and Phase II studies of pyrotinib demonstrated that pyrotinib had good safety and promising antitumor activity in patients with metastatic breast cancer that is HER2‐positive.^7^, ^18^ Subgroup analyses of these studies suggest the favourable efficacy of pyrotinib in patients with HER2‐positive breast cancer, regardless of whether they had previously been treated with trastuzumab.

In first‐line treatment, trastuzumab, pertuzumab, and docetaxel in the CLEOPATRA study greatly extended the OS in patients with breast cancer that is HER2‐positive, with an ORR of 80.2%. However, the proportion of the trastuzumab‐treated population in the study was only 11%.^4^ The PHILA study, a phase III randomized trial of trastuzumab and docetaxel plus pyrotinib or placebo for HER2‐positive metastatic breast cancer, reported an ORR of 83% and an mPFS of 24.3 months in the pyrotinib group (10.4 months in the placebo group, hazard ratio = .41),^19^ but the proportion of the trastuzumab‐treated patients in the pyrotinib group was also relatively low at around 15.5%. The subgroup analysis of PHILA study showed greater PFS benefit in patients who have received adjuvant and/or neoadjuvant trastuzumab (not reached versus 9.3 months; hazard ratio = .23) compared to those who had not (21.9 vs. 10.4 months; hazard ratio = .45). Similarly, the PANDORA study, a phase II single‐arm trial of pyrotinib plus docetaxel as first‐line treatment for metastatic breast cancer that is HER2‐positive, reached an ORR of 79.7%, with 30.4% of the patients had previously received trastuzumab therapy.^20^ The subgroup analysis revealed that patients who have received previous (neo)adjuvant trastuzumab therapy exhibited a longer mPFS than patients who had not (20.8 vs. 14.8 months). However, these studies did not adequately represent the prevailing clinical reality, as they lacked sufficient representation of patients who have received previous trastuzumab therapy. Compared with these studies, our study demonstrated a notably higher ORR value, indicating that the combination of pyrotinib and nab‐PTX exhibits remarkable efficacy in patients who have received adjuvant and/or neoadjuvant trastuzumab therapy and may serve as a viable alternative when trastuzumab resistance develops.

The concept of trastuzumab primary resistance was proposed in 2011.^16^ It is believed that further anti‐HER2 treatment strategy should be developed according to whether the patient is trastuzumab primary‐resistant or not, but the exploration of first‐line treatment options after trastuzumab resistance in research remains lacking. The PHOEBE study, which investigated the efficacy of pyrotinib or lapatinib plus capecitabine in the second‐line treatment for HER2‐positive metastatic breast cancer, presented additional analyses of the trastuzumab‐resistant subgroup at the San Antonio Breast Cancer Symposium in 2021. The study demonstrated a significant PFS benefit in the pyrotinib group, regardless of trastuzumab resistance. Trastuzumab resistance was defined as recurrence within 6 months of adjuvant trastuzumab therapy and progression within 3 months of trastuzumab therapy for metastatic breast cancer. There were 42.5% of patients had not received first‐line treatment in the pyrotinib group. Based on the three definitions of trastuzumab resistance,^21^ the PICTURE study, a phase II trial of pyrotinib plus capecitabine for HER2‐positive advanced breast cancer that is trastuzumab‐resistant, also performed a subgroup analysis and found that patients who progressed within 12 months after completion of adjuvant/neoadjuvant trastuzumab therapy had the longest mPFS of 17.8 months compared with those who progressed during adjuvant/neoadjuvant trastuzumab therapy and those who progressed within 6 months of initiating trastuzumab therapy in the advanced stage. In this subgroup, 66% of patients lacked first‐line treatment.^22^ In our study, the mPFS of patients who progressed within 12 months after completion of adjuvant/neoadjuvant trastuzumab therapy reached 18.3 months, which was slightly higher than the PICTURE study. Unlike the subgroup analyses of primary resistance in these studies, our study initially explored the impact of primary and secondary trastuzumab resistance on the efficacy of the pyrotinib regimen. Although our results did not show a significant difference, it is possible that the limited enrollment of the patients in our study may have contributed to this outcome.

Other subgroup analyses in our study showed that patients with non‐visceral metastases had longer PFS than those with non‐visceral metastases. The site of metastasis has been shown in several studies to be one of the most important factors affecting prognosis.^23^, ^24^ Our results confirmed this finding. However, As a result of a limited number of patients, dual‐targeted therapy subgroups were unable to be further analyzed. This may be due to the higher cost of dual‐targeted therapy or the significant reduction in the recurrence rate of breast cancer attributed to dual‐targeted therapy.

The predominant adverse effect in this study was diarrhea, aligning with the common adverse effect associated with tyrosine kinase inhibitors targeting epidermal growth factor receptors.^7^, ^25^ In this study, diarrhoea was prevalent in all patients, with 29.6% of patients experiencing grade 3 diarrhea, comparable to findings in previous investigations involving pyrotinib.^8^, ^9^, ^19^, ^22^ Severe diarrhoea typically manifested during the initial phase of treatment but was ameliorated through anti‐diarrheal interventions, treatment interruption, or dose reduction. As treatment progressed, the incidence of diarrhoea decreased gradually and the symptoms ameliorated compared with the previous period. Loperamide and montmorillonite were effective in relieving diarrhea. Haematological adverse events, predominantly attributed to nab‐PTX, constituted a significant proportion of grade 3 and higher AEs. By using leukopoietic agents, the haematological adverse events induced by nab‐PTX can be effectively managed. After the discontinuation of nab‐PTX, a notable decrease in the incidence of hematologic toxicities was observed in patients. In comparison to other clinical trials involving the same drugs, our study found that the incidence of adverse effects was not greater and could be effectively managed.

Dynamic analyses showed the downregulation of several proteins during treatment, with CEACAM5, TXLNA, PVRL4, and ERBB2 being the most significant. CEACAM5, also known as carcinoembryonic antigen (CEA), has been reported to be overexpressed in several cancers, especially colon cancer.^26^, ^27^, ^28^ It participates in the inhibition of cell differentiation and apoptosis,^29^ and plays an important role in tumour metastasis.^30^ A significant association was found between elevated CEA levels and tumour size, lymph node metastasis, and advanced‐stage breast cancer.^31^, ^32^, ^33^ Studies have found that CEA levels were affected by drugs such as trastuzumab and paclitaxel.^33^, ^34^ Alpha‐taxilin (TXLNA) has been reported in renal cell cancer, hepatocellular cancer, and pancreatic cancer.^35^, ^36^, ^37^ To our knowledge, no study has found an association between TXLNA and breast cancer. Nectin‐4 (PVRL4) is expressed in cancer tissues such as breast cancer. PVRL4 has been demonstrated to have an important effect on tumor growth, metastasis, and drug resistance.^38^, ^39^ In breast cancer, PVRL4 was demonstrated to activate the WNT/β‐Catenin signalling pathway via the Pi3k/Akt axis, consequently controlling the proliferation of cancer stem cells.^40^ We speculate that the reduction of these markers over the treatment course may be associated with the effectiveness of the combination. Further exploration of the possible mechanisms of changes in these markers may lead to a better interpretation of the mechanism of drug resistance in tumours.

Survival‐associated proteomic analyses showed that patients with a high level of TLR3 and a low level of RET at the baseline node had longer PFS. In the immune system, TLR3 plays a crucial role.^41^ In breast cancer, TLR3 could inhibit tumor proliferation by interacting with the GFR/PI3K/AKT pathway, suggesting that TLR3 is a potential repressor for breast cancer initiation and progression.^42^ Also, triple‐negative breast cancer shows a better prognosis when TLR3 is highly expressed.^43^ In the immunotherapy, it was found that TLR3 agonists could activate the anti‐tumor immune responses based on Killer T Cells.^44^, ^45^, ^46^ In addition, tumor cells without TLR3 have been shown to be more resistant to chemotherapy.^47^ Our results further support these conclusions, while in other studies, activation of TLR3 promotes the transformation of breast cancer cells to cancer stem cells, leading to the acquisition of stem cell properties.^48^, ^49^ As for the dual role of TLR3, it has been found that pro‑ and antitumor effects have been observed in the same cell line depending on the mode of delivery of the TLR3‐ligand, with surface stimulation leading to protumoural effects whereas cytoplasmic stimulation has been antitumoral in two breast cancer cell lines.^50^ We speculate that TLR3 is a favorable factor in our study, which may be related to the stimulation of TLR3 through cytoplasm. More samples need to be collected to validate this result, and the molecular mechanisms need to be further explored. Activation of the MAPK, PI3K, and STAT pathways has been widely acknowledged to drive cell growth and division in various cancers, including breast cancer. RET plays a crucial role in mediating these intracellular signalling pathways.^51^ Mutations of the RET gene cause abnormal RET expression, subsequently initiating and advancing tumour development. RET mutations have been identified in several cancer types, with thyroid cancer and lung cancer having the highest prevalence.^52^, ^53^ Now RET has become an important therapeutic target.^54^ In addition to its involvement in the initiation and progression of breast cancer,^55^ studies found that RET was associated with resistance to endocrine therapy.^56^ Our results are consistent with the effects of RET in promoting cancer cell proliferation and invasion, as patients with high RET expression demonstrated shorter PFS.

Diarrhoea is a common adverse event associated with TKIs. However, the exact mechanism underlying TKI‐induced diarrhoea remains unclarified and may be multifactorial, including factors such as ion transport dysregulation, mucosal injury, and inflammation.^57^ Our results showed that compared with patients with grade I and II diarrhea, CRNN was significantly upregulated at baseline in patients with grade III diarrhea. It is worth noting that CRNN is downregulated in various epithelial squamous cell cancers of the head and neck, oesophagus, and cervix.^58^ Dysregulation of CRNN has also been observed in various skin diseases, including eczema.^59^ To date, no studies have reported any association with TKIs or diarrhea. Therefore, further exploration is needed to understand the potential mechanisms underlying the proteomic findings in our study.

The study has several limitations, including a small sample size and a single‐arm design without a control group. Results could be optimized by enrolling more patients.

## 5 CONCLUSION

This trial demonstrates that the combination of pyrotinib and nab‐PTX as a first‐line treatment regimen delivers favourable efficacy with manageable safety in patients with metastatic breast cancer that is HER2‐positive who have previously received adjuvant and/or neoadjuvant trastuzumab therapy. In addition, our results suggest that TLR3 and RET might serve as potential predictive proteins for PFS in these patients. However, further investigation is warranted to validate these findings in larger cohorts and to elucidate the underlying mechanisms of differential proteins and survival‐associated proteins.

## AUTHOR CONTRIBUTIONS

Study design, supervision, funding acquisition, methodology: Huihui Li. Provision of study materials or patients: Dongdong Zhou, Baoxuan Zhang, Shu Fang, Fangchao Zheng, Chao Li, Xinzhao Wang, Wei Huang, Linlin Wang, Qingqing He, Hui Fu, Yan Zhang, Changrui Liu, Lin Dong, Xianguang Zhao, Liang Xu, Xiao Sun, Bingjie Fan, Lihua Song, Zhengbo Zhou, and Jinming Yu. Data collection and analysis: Xiaochu Man, Jie Huang, and Shujuan Sun. Manuscript writing and review: Xiaochu Man, Jie Huang, and Shujuan Sun. All authors read and approved the final version of the manuscript.

## CONFLICT OF INTEREST STATEMENT

The authors declare no conflict of interest.

## ETHICS STATEMENT

The study was conducted in accordance with the Declaration of Helsinki and Good Clinical Practice guidelines. The protocol and all amendments were approved by the ethics committee of Shandong Cancer Hospital (SDZLEC2019‐056‐01). All patients provided written informed consent.

## CONSENT FOR PUBLICATION

All participants have agreed to publish information about their treatment and other reports in this study after keeping their private information confidential.

## Supporting information

Supporting information

Supporting information

Supporting information

Supporting information

Supporting information

Supporting information

Supporting information

Supporting information

## Data Availability

The datasets used and analyzed during the current study are available from the corresponding author upon reasonable request.
