# Supplementary figures and images for: Efficacy and safety of pyrotinib combined with albumin‐bound paclitaxel as first‐line treatment for HER2‐positive metastatic breast cancer in patients previously treated with adjuvant and/or neoadjuvant trastuzumab therapy: The stage 1 results of a single‐arm, phase 2 prospective clinical trial
Source: Clin Transl Med. 2024 May 13;14(5):e1687. doi: 10.1002/ctm2.1687 (PMC11089842; doi:10.1002/ctm2.1687)

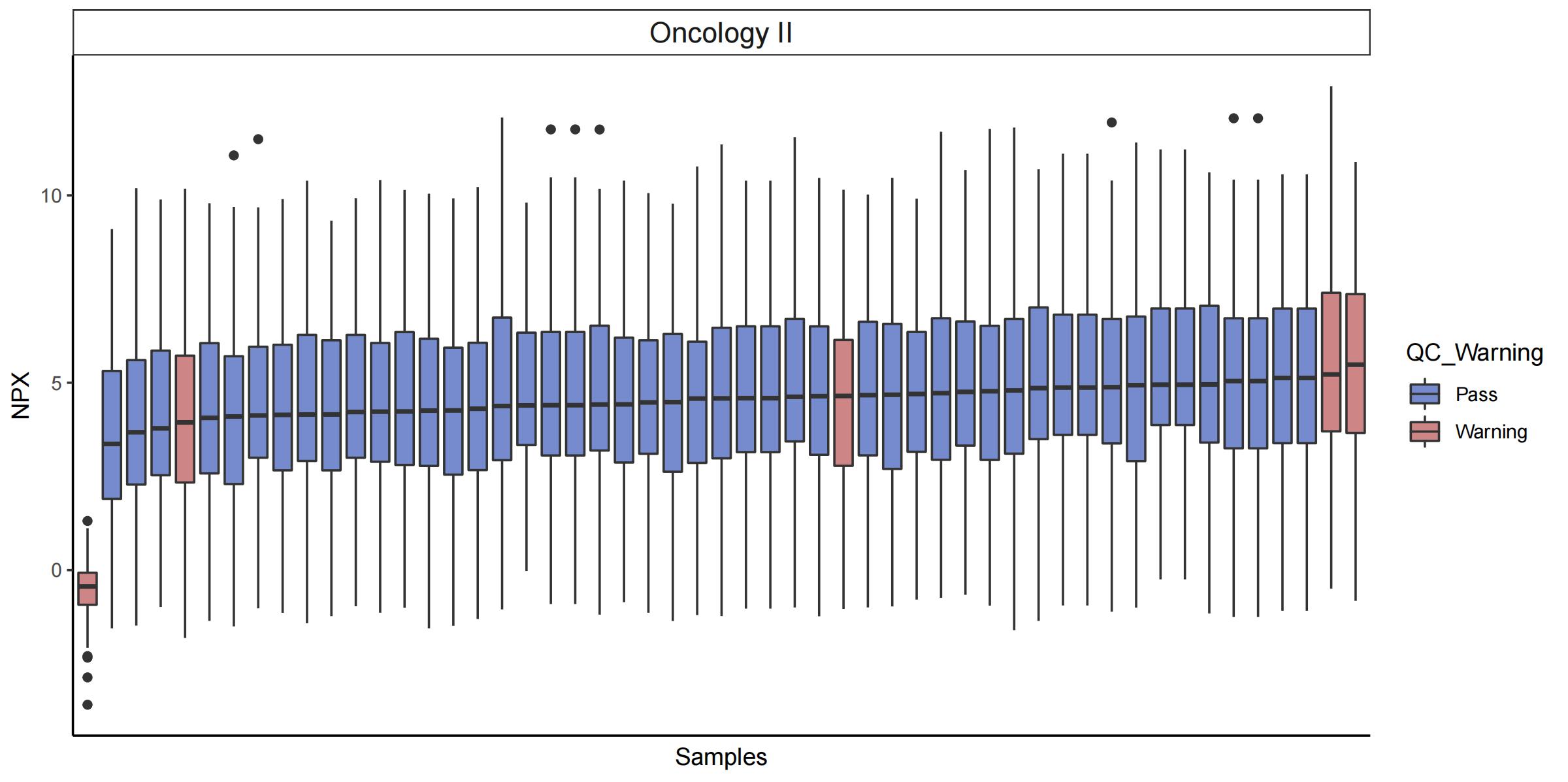

Supplement: Supplementary file 1 — Supporting information [file CTM2-14-e1687-s003.jpg]

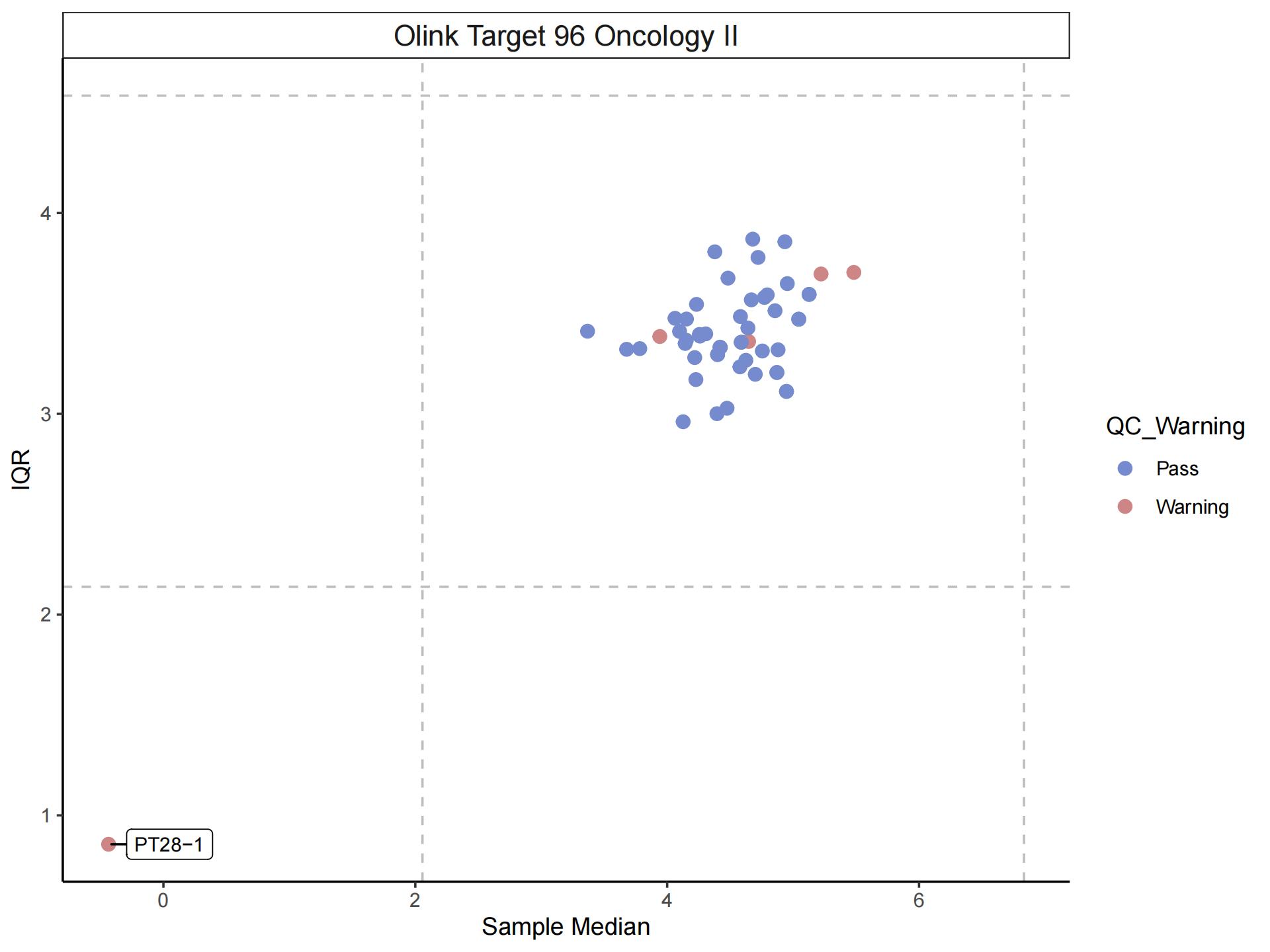

Supplement: Supplementary file 2 — Supporting information [file CTM2-14-e1687-s002.jpg]

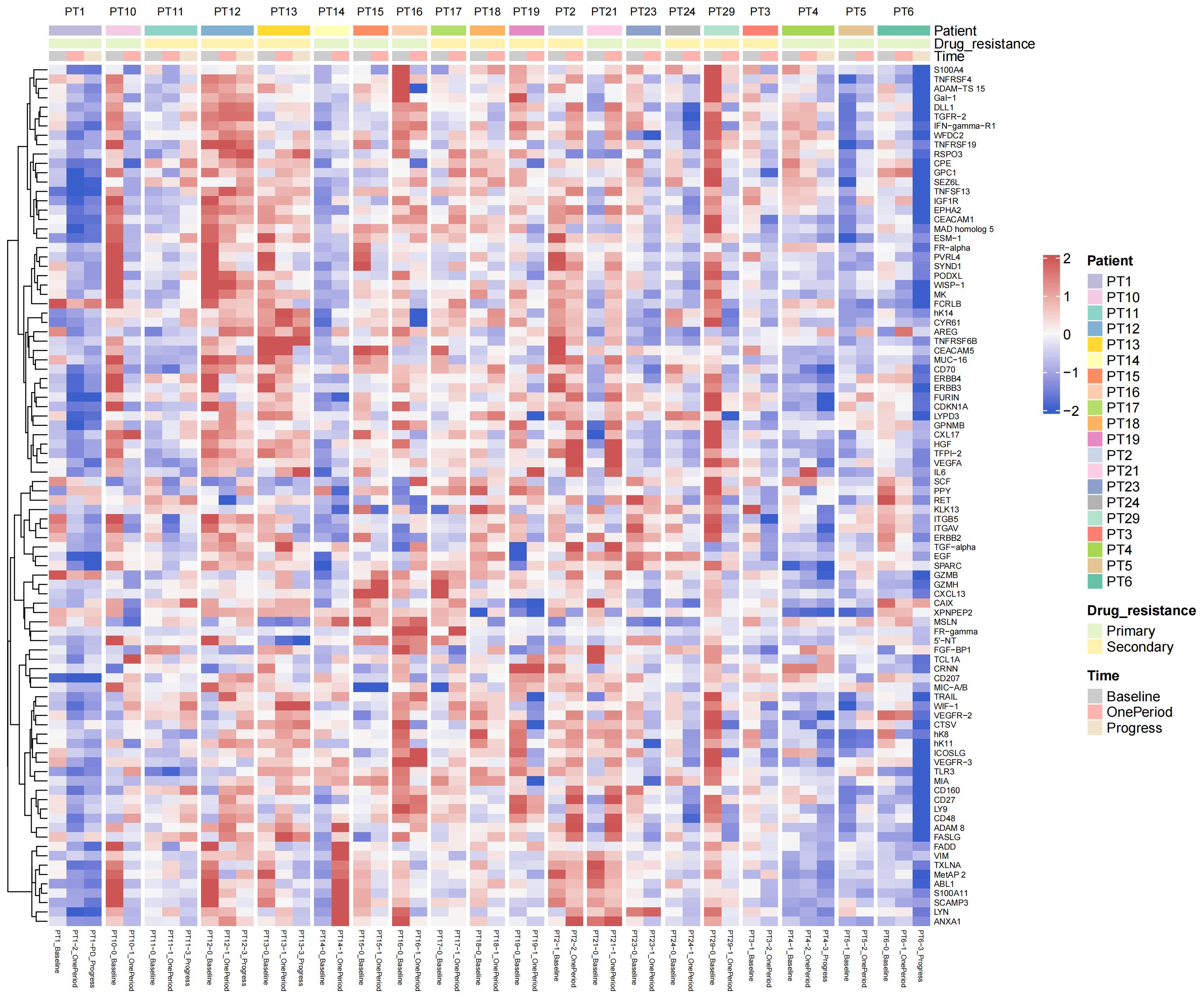

Supplement: Supplementary file 3 — Supporting information [file CTM2-14-e1687-s007.jpg]

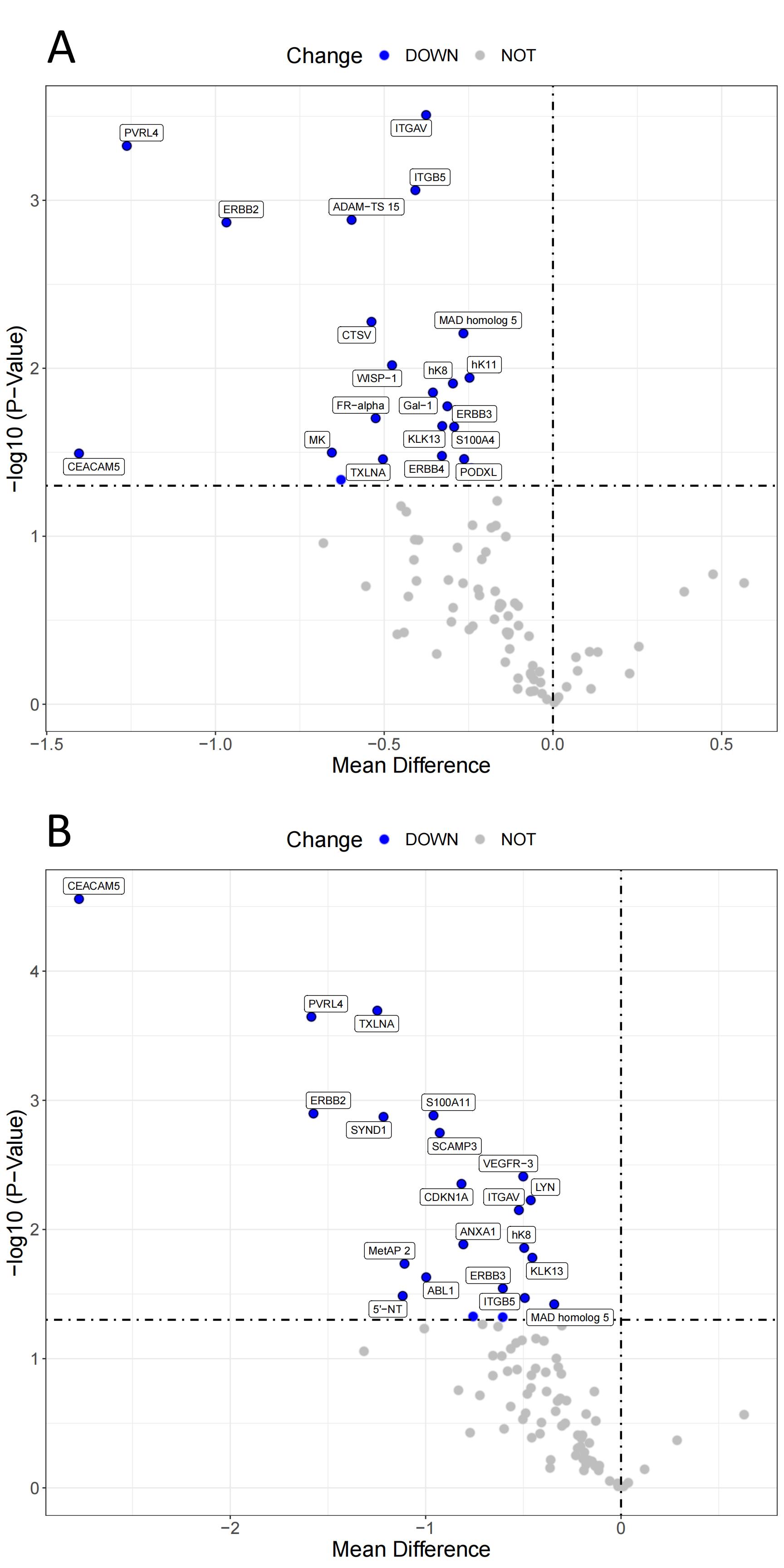

Supplement: Supplementary file 4 — Supporting information [file CTM2-14-e1687-s005.jpg]

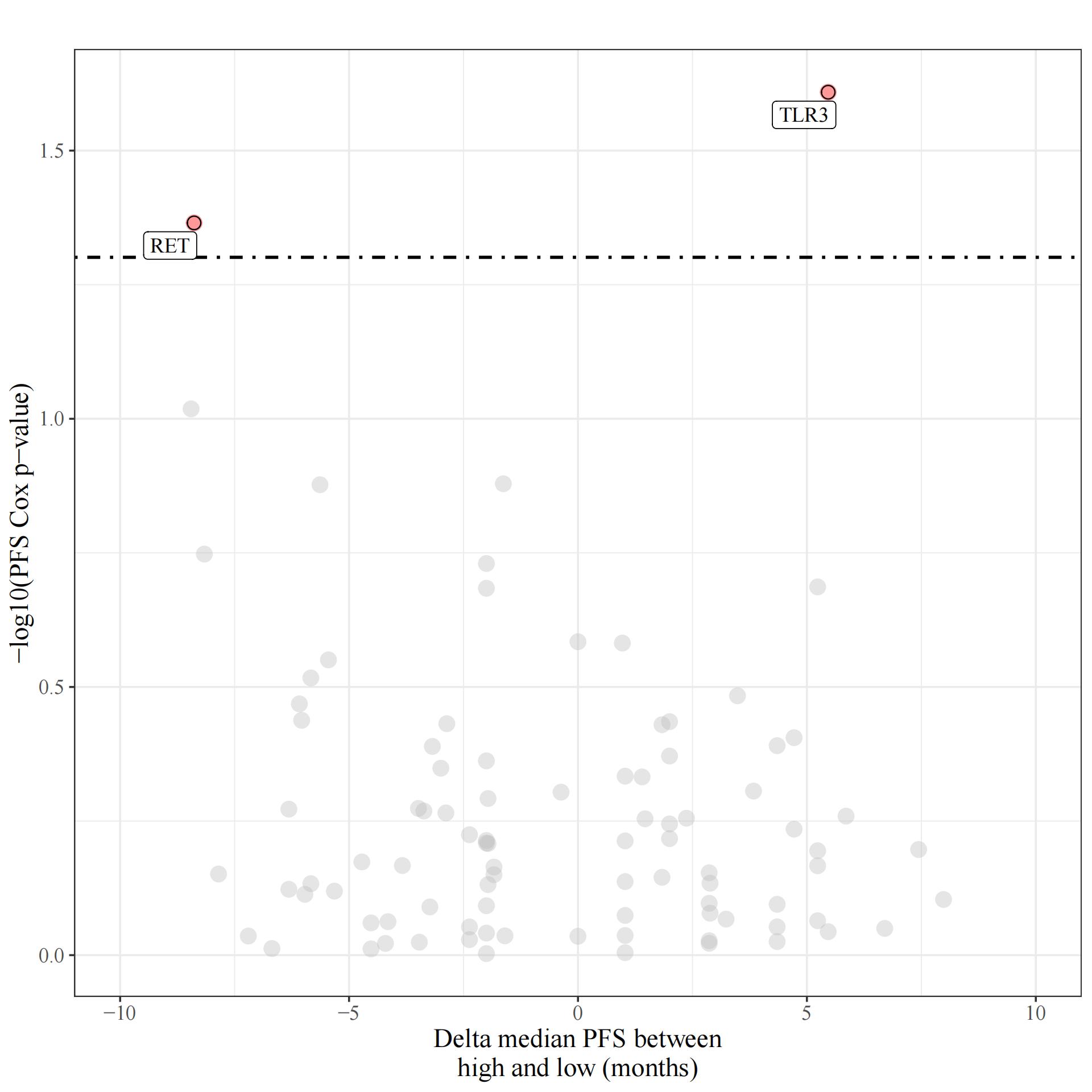

Supplement: Supplementary file 5 — Supporting information [file CTM2-14-e1687-s001.jpg]

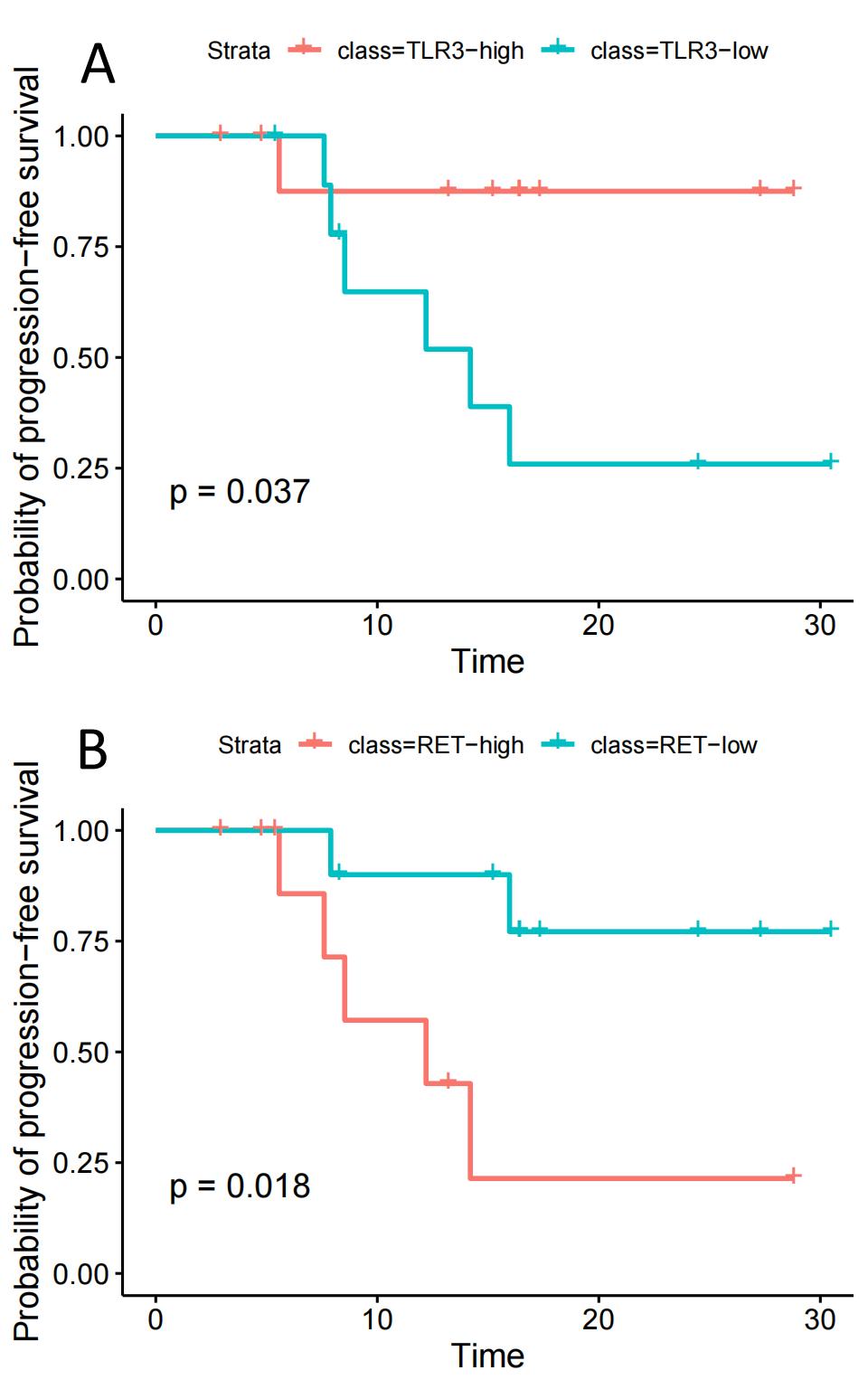

Supplement: Supplementary file 6 — Supporting information [file CTM2-14-e1687-s008.jpg]

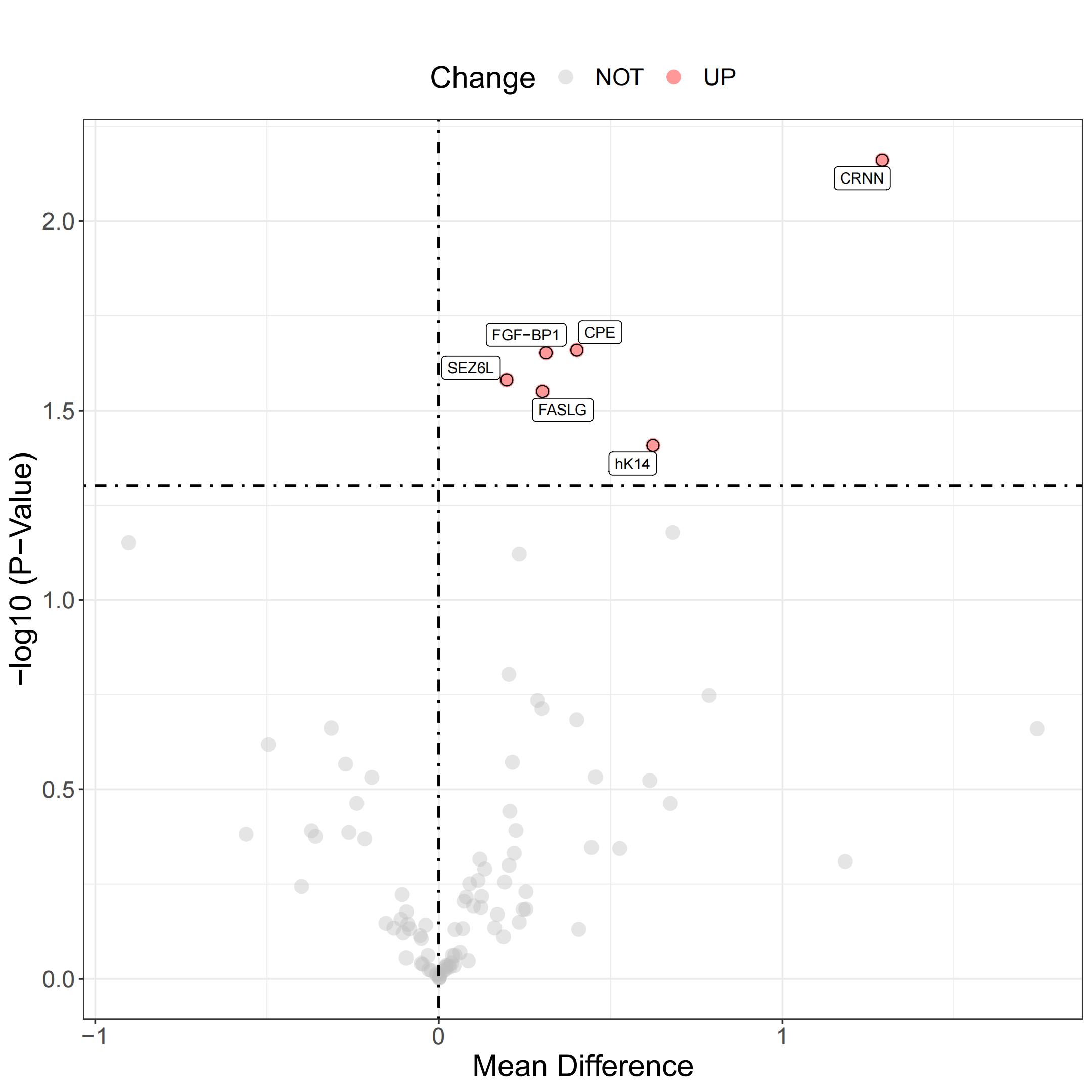

Supplement: Supplementary file 7 — Supporting information [file CTM2-14-e1687-s004.jpg]
