# Supplementary material for: Efficacy and safety of pyrotinib combined with albumin‐bound paclitaxel as first‐line treatment for HER2‐positive metastatic breast cancer in patients previously treated with adjuvant and/or neoadjuvant trastuzumab therapy: The stage 1 results of a single‐arm, phase 2 prospective clinical trial
Source: Clin Transl Med. 2024 May 13;14(5):e1687. doi: 10.1002/ctm2.1687 (PMC11089842; doi:10.1002/ctm2.1687)
Supplement: Supplementary file 8 — Supporting information [file CTM2-14-e1687-s006.docx]

**Figure legends**

**Figure S1.** NPX values.

**Figure S2.** Interquartile Range.

**Figure S3.** Heat map of overall protein expression.

**Figure S4.** (a) Dynamic proteomic analyses showed 23 proteins were downregulated at the pre-therapy of the second cycle compared to the baseline. (b) Dynamic proteomic analyses showed 22 proteins were downregulated at the progression compared to the baseline.

**Figure S5.** Survival-associated proteomic analyses. RET and TLR3 proteins at baseline were significantly associated with PFS in patients who received pyrotinib plus nab-PTX as a first-line treatment regimen.

**Figure S6.** (a) KM survival analysis of TLR3. (b) KM survival analysis of RET.

**Figure S7.** Grade III diarrhea associated proteomic analyses. Compared to patients with diarrhea grade I and II, patients with grade III diarrhea had 6 proteins upregulated at baseline.
